# Diffractive Trifocal Intraocular Lens Implantation in Stable Subclinical and Forme Fruste Keratoconus: A Case Series

**DOI:** 10.7759/cureus.39134

**Published:** 2023-05-17

**Authors:** Abdulhameed H Mahmood, Anoud F Alsaati

**Affiliations:** 1 Ophthalmology, Salmaniya Medical Complex, Manama, BHR; 2 Ophthalmology, King Faisal Specialist Hospital and Research Centre, Riyadh, SAU

**Keywords:** trifocal iols, corneal topography, forme-fruste keratoconus, premium iol, diffractive iol, tri-focal iol, cataract, keratoconus (kc), cornea

## Abstract

In this retrospective case series, we examine the outcomes of diffractive trifocal intraocular lens implantation after cataract surgery, in patients with subclinical and forme fruste keratoconus. Eight eyes of four patients (aged between 47 and 64) were included in the study and underwent phacoemulsification with implantation of AT LISA tri 839MP or AT LISA tri-toric 939MP intraocular lenses (Carl Zeiss Meditec AG, Jena, Germany). Post-operative evaluation included a visual acuity test at three distances (6m, 80cm, and 40cm), a visual acuity test at three low contrast levels (25%, 12.5%, and 6%), and a questionnaire about the patients' experience with photic phenomena and overall satisfaction with the achieved quality of vision. Our results show that spectacle freedom was achieved in all cases with a high satisfaction rate among participants. We hope our results would encourage surgeons to offer this technology to carefully selected candidates with stable subclinical and forme fruste keratoconus undergoing cataract surgery, giving them the possibility of achieving spectacle independence.

## Introduction

Since the debut of the first generation of multifocal intraocular lenses (IOLs) in the late 1980s, the technology of presbyopia-correcting IOLs has gone through several milestones that revolutionized cataract surgery, the last of which was the introduction of tri-focal and extended depth of focus (EDOF) IOL designs to improve intermediate vision, which had been acknowledged by patients as a reason for the quality of life impairment among pseudophakics [1,2]. Given the negative impact of corneal astigmatism, coma, and other high-order aberrations on the performance of trifocal IOLs, patients with suspected keratoconus or those with forme fruste keratoconus have classically been considered poor candidates for this technology [3-5].

Several case reports and case series have reported encouraging results after the implantation of toric IOLs in keratoconus patients [6-13], but only two previous studies presented the outcomes of toric trifocal IOLs in these patients. Montano et al. [14] reported two cases of stable forme fruste keratoconus and cataract, with good visual outcomes after trifocal IOL implantation and Farideh et al. [15] published the results of 10 eyes (five patients) with stable mild keratoconus and cataract, and concluded that the implantation of toric trifocal IOLs in these selected cases provided satisfactory visual outcome in all three distances. 

Previous evidence shows that keratoconus patients, in general, have better tolerance to defocus than normal patients, allowing them to better tolerate residual refractive errors after IOL implantation [16]. In 2018, Lisa et al. reported good visual and refractive outcomes of 17 eyes (11 patients) with stable keratoconus, who underwent sequential intrastromal corneal ring segment implantation, followed by cataract extraction six months later, with implantation of extended depth of focus IOL, thus taking advantage of these patients’ better tolerance to defocus, compared to healthy individuals [17].

Based on the encouraging results of previous studies, particularly those published by Montano et al. [14] and Farideh et al. [15], it can be concluded that offering the trifocal IOL option for selected cases with stable early keratoconus, or those with borderline corneal topography, is still possible, especially when taking into consideration other factors such as the absence of ocular surface disease, the patient’s motivation for spectacle independence, and meticulous IOL power estimation.

In this paper, we report a series of four patients (eight eyes) with stable subclinical and forme fruste keratoconus and cataract, who underwent sequential bilateral phacoemulsification and implantation of diffractive trifocal IOLs, with excellent post-operative visual outcomes and patient satisfaction after a minimum of one-year follow-up.

This article was previously presented as a meeting abstract at the Saudi Ophthalmology 2021 Virtual Conference on March 20, 2021.

## Case presentation

In this retrospective non-comparative case series, eight eyes of four patients who were considered to be cases of subclinical or forme fruste keratoconus were included, with a mean age of 54.2 (ranging from 47 to 64). Inclusion criteria included patients with cataract, positive family history of keratoconus, and a Scheimpflug-based corneal tomography test displaying at least one of the following findings: (1) Inferior steepening on keratometry map, (2) A suspiciously high value on the anterior elevation map (>8), (3) A suspiciously high value on the posterior elevation map (>18), (4) An abnormal deviation of the thinnest location point (more than 1mm in the horizontal (x) axis, or more than 0.5mm in the vertical (y) axis) on the pachymetry map, (5) A difference between corneal thickness at the thinnest location and corneal thickness at the apex of more than 10µm. Patients with clinical keratoconus and any other ocular comorbidities were excluded from the study.

Table 1 shows corneal tomography parameters of all cases, with bold and italic numbers denoting suspicious values and Figures 1-8 show the four-map refractive display of all the eyes included, obtained using Scheimpflug-based tomography systems.

**Table 1:** Corneal tomography parameters of included cases I-S: Inferior-Superior Keratometric Asymmetry Index; TL: Thinnest Location; BFS: Best-Fit Sphere; BAD-D: Belin-Ambrosio Display overall D-Value; RMS–HOA @ 6mm: Root Mean Square of the total Corneal Higher Order Aberrations within the 6mm pupil. NOTE: Suspicious values in bold and Italic

Parameter	Eye	Case 1	Case 2	Case 3	Case 4
Keratometry (I – S)	Right Eye	0.9D	0.9D	1.9D	1D
	Left Eye	0.9D	1.2D	2.5D	0.9D
Pachymetry	Right Eye	567µm	576µm	525µm	540µm
	Left Eye	554µm	564µm	509µm	529µm
Deviation of TL	Right Eye	x:-0.89, *y:-0.82 *	*x:-1.14*, y:-0.20	x:-0.67, *y:-0.61 *	x:-0.76, *y:-0.82 *
	Left Eye	x:+1.24, y:-0.89	*x:+1.38*, y:0.0	x:+0.64, *y:-1.80*	x:+0.99, *y:-0.77*
Pachy Apex – TL	Right Eye	10µm	13µm	5µm	8µm
	Left Eye	18µm	16µm	23µm	9µm
Anterior Corneal Elevation (BFS)	Right Eye	+5µm	+4µm	*+12µm *	+2µm
	Left Eye	+6µm	+4µm	+20µm	+2µm
Posterior Corneal Elevation (BFS)	Right Eye	+22µm	+20µm	*+35µm *	*+26µm *
	Left Eye	+27µm	+16µm	+49µm	+24µm
BAD –D	Right Eye	1.46	0.97	1	2.14
	Left Eye	1.85	0.68	5.49	2.16
RMS- HOA @ 6mm	Right Eye	0.386µm	0.758µm	0.883µm	1.27µm
	Left Eye	0.475µm	0.738µm	1.25µm	1.26µm

**Figure 1:**
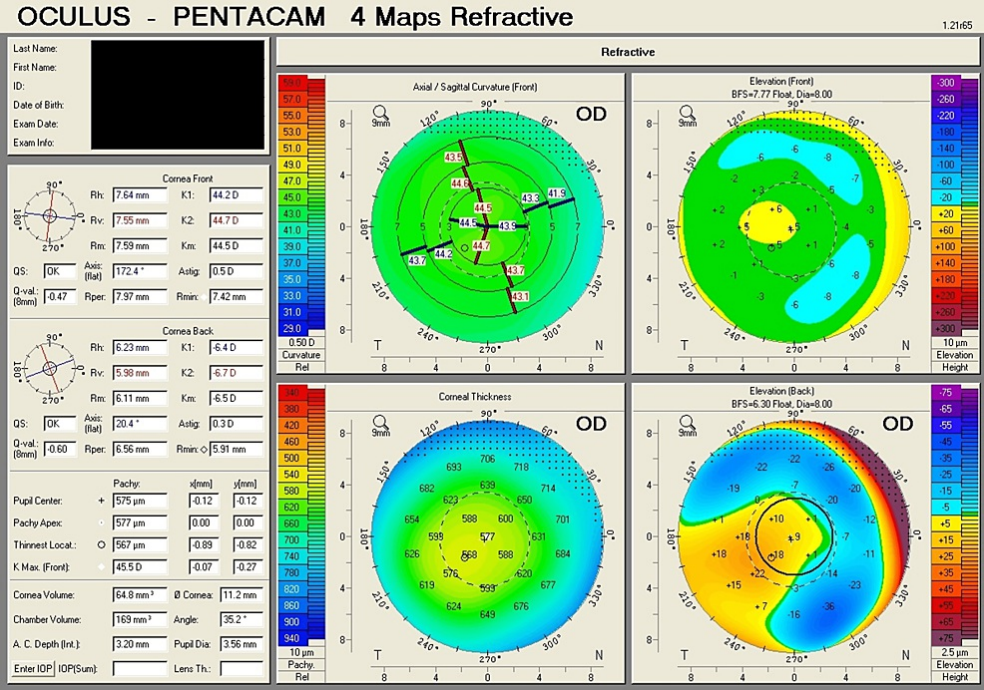
Right eye corneal tomography image (four-map refractive display) of Case 1

**Figure 2:**
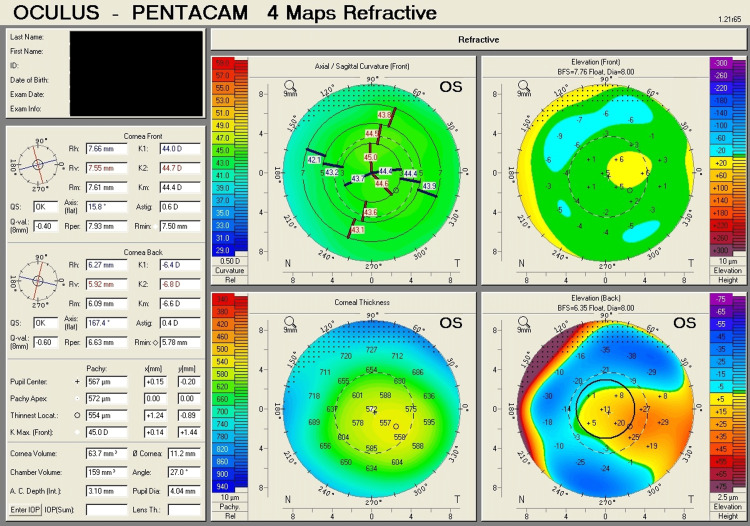
Left eye corneal tomography image (four-map refractive display) of Case 1

**Figure 3:**
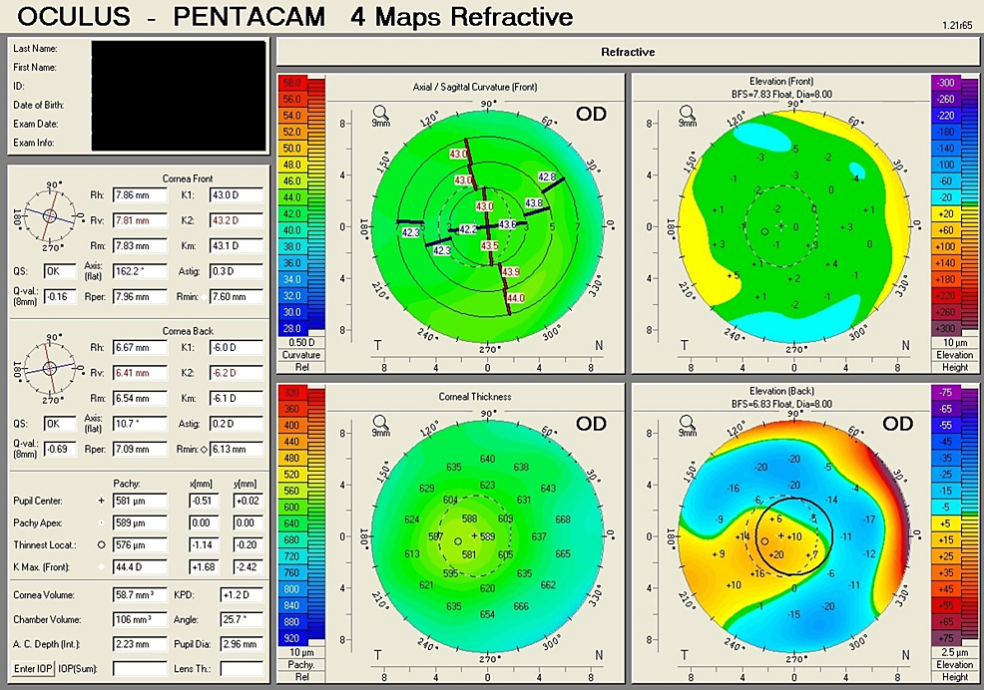
Right eye corneal tomography image (four-map refractive display) of Case 2

**Figure 4:**
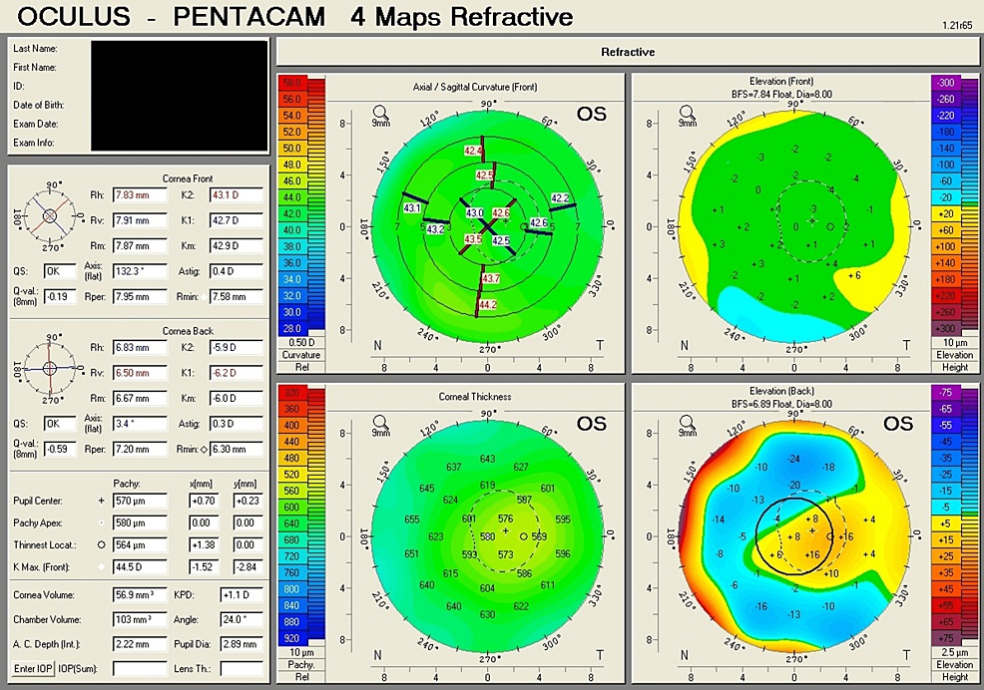
Left eye corneal tomography image (four-map refractive display) of Case 2

**Figure 5:**
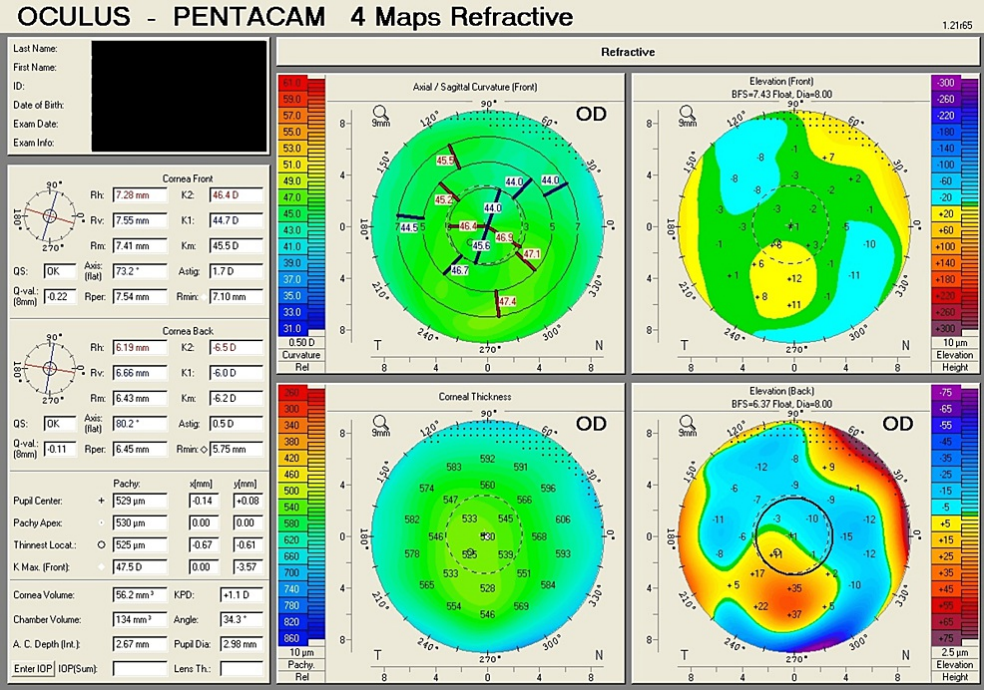
Right eye corneal tomography image (four-map refractive display) of Case 3

**Figure 6:**
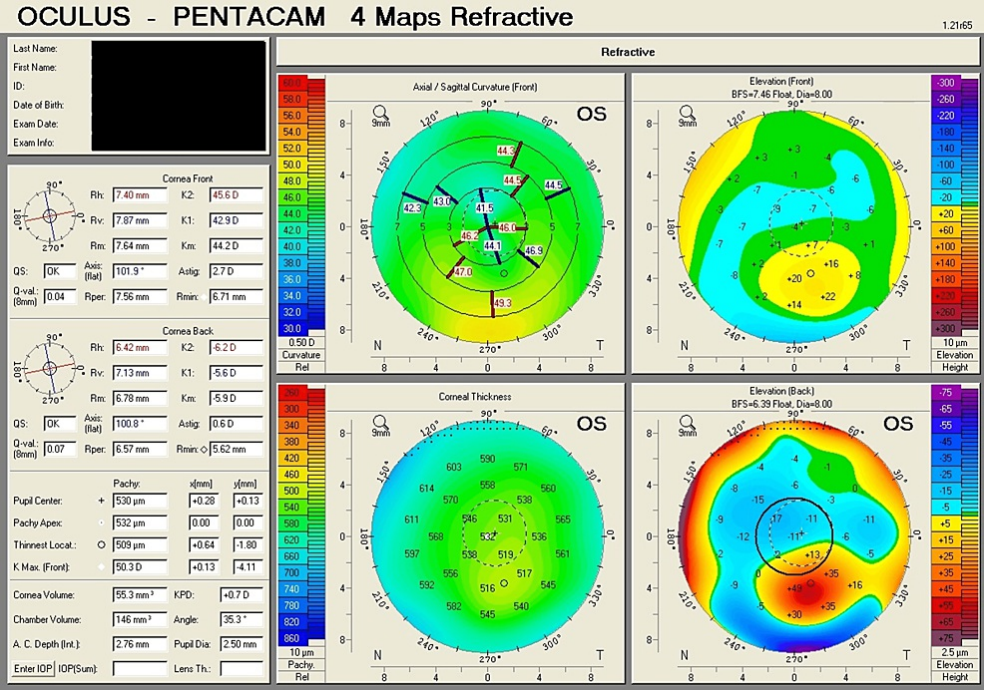
Left eye corneal tomography image (four-map refractive display) of Case 3

**Figure 7:**
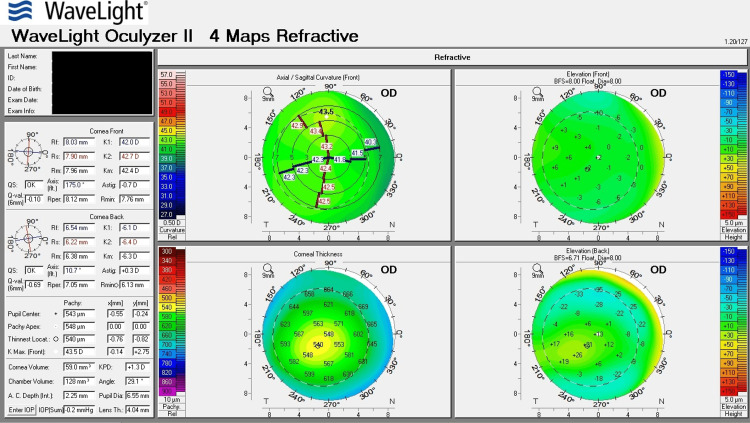
Right eye corneal tomography image (four-map refractive display) of Case 4

**Figure 8:**
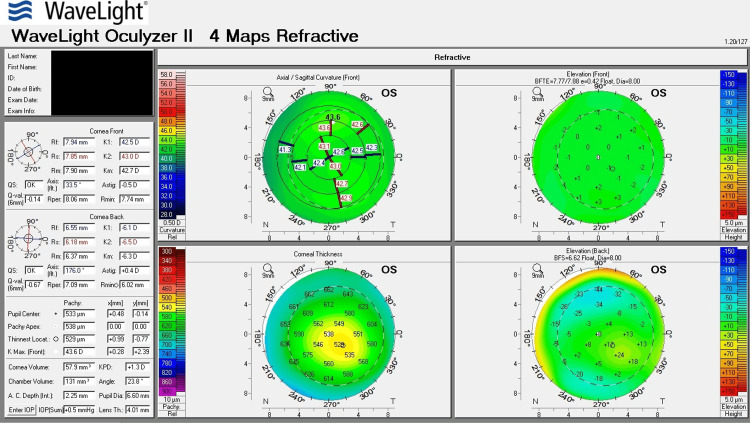
Left eye corneal tomography image (four-map refractive display) of Case 4

IOL selection

Six eyes (three patients) received the non-toric AT LISA tri 839MP IOL (Carl Zeiss Meditec AG, Jena, Germany), while two eyes of case 3 in the series received the toric version of the IOL, AT LISA tri-toric 939MP (Carl Zeiss Meditec AG), because corneal astigmatism was higher than 1.5D in both eyes.

The AT LISA tri family is a group of diffractive, pupil-independent, trifocal IOLs that consist of an optical zone that has a 3.33 D near addition and a 1.66 D intermediate addition. They are designed to transmit 85.7% of incident light with an asymmetric distribution of 50%, 20%, and 30% for far, intermediate, and near foci, respectively [18].

Both AT LISA tri 839MP and AT LISA tri-toric 939MP are of a single-piece design, made of a hydrophilic acrylic material (25%) with hydrophobic surface properties. The optic diameter is 6.0mm with a total IOL diameter of 11.0mm [18].

Surgery

All surgeries were performed by one surgeon (AFA) following the same protocol. Standard technique phacoemulsification under topical anesthesia was performed. Adequate pupil dilatation was obtained using preoperative dilating eye drops and intracameral mydriasis. The IOL was implanted in all cases inside the capsular bag, and proper axis alignment was ensured for those with toric IOL implants. No complications occurred during the surgeries. Post-operative topical therapy regimen included an antibiotic eye drop (Moxifloxacin 0.5%, four times a day for one week) and a steroid eye drop (Prednisolone acetate 1%, four times a day for the first week, with weekly tapering).

Follow-up

All patients were seen on post-opertive day 1 and day 30 and were then given routine follow-up appointments. The final assessment was done one year after the second eye surgery. At the final assessment, all patients answered a questionnaire regarding their satisfaction with the final outcomes of the surgery, and their experience with photic phenomena after the surgery.

All patients underwent a fully functional visual assessment, which included visual acuity at all three distances (Far at 6 meters, Intermediate at 80 centimeters, and Near at 40 centimeters). Moreover, contrast sensitivity was evaluated by testing visual acuity at three low-contrast levels (25%, 12.5%, and 6%).

Visual acuity

All patients achieved 20/20^-2^ (logMAR 0.08) or better for uncorrected near visual acuity and 20/30 (logMAR 0.18) or better for uncorrected intermediate visual acuity. While cases 1, 2, and 4 all achieved an uncorrected distance visual acuity of 20/20^-2^ (logMAR 0.08) or better, Case 3 recorded a distance visual acuity of 20/40 (logMAR 0.3) in the right eye and 20/30 (logMAR 0.18) in the left eye, and this was due to a myopic post-operative refractive outcome of -0.75D. However, the patient was still satisfied with the overall outcome and did not require spectacle correction. Table 2 shows the detailed visual acuity results of all cases included at all distances.

**Table 2:** Post-operative visual acuity values at far (6m), near (40cm), and intermediate (80cm) distances logMAR: logarithm of the minimum angle of resolution

Distance	Eye		Case 1	Case 2	Case 3	Case 4
Far		Right Eye	20/20^-2^ (logMAR 0.08)	20/20 (logMAR 0)	20/40 (logMAR 0.3)	20/20 (logMAR 0)
		Left Eye	20/20 (logMAR 0)	20/20 (logMAR 0)	20/30 (logMAR 0.18)	20/20^-2^ (logMAR 0.08)
Intermediate		Right Eye	20/20 (logMAR 0)	20/20 (logMAR 0)	20/30 (logMAR 0.18)	20/30 (logMAR 0.18)
		Left Eye	20/20 (logMAR 0)	20/30 (logMAR 0.18)	20/30 (logMAR 0.18)	20/20 (logMAR 0)
Near		Right Eye	20/20 (logMAR 0)	20/20 (logMAR 0)	20/20^-1^ (logMAR 0.04)	20/20^-2^ (logMAR 0.08)
		Left Eye	20/20 (logMAR 0)	20/20 (logMAR 0)	20/20^-2^ (logMAR 0.08)	20/20 (logMAR 0)

Contrast sensitivity

All eyes achieved a visual acuity of 20/30 (logMAR 0.18) or better at contrast levels of 25% and 12.5%, and a visual acuity of 20/40 (logMAR 0.3) or better at a contrast level of 6% as shown in Table 3.

**Table 3:** Post-operative visual acuity at low contrast levels (25%, 12.5%, and 6%) logMAR: logarithm of the minimum angle of resolution

Contrast Level	Eye	Case 1	Case 2	Case 3	Case 4
25%	Right Eye	20/25 (logMAR 0.097)	20/20 (logMAR 0)	20/20 (logMAR 0)	20/20 (logMAR 0)
	Left Eye	20/22.5 (logMAR 0.05)	20/20 (logMAR 0)	20/28.5 (logMAR 0.15)	20/25 (logMAR 0.097)
12.5%	Right Eye	20/28.5 (logMAR 0.15)	20/25 (logMAR 0.097)	20/25 (logMAR 0.097)	20/25 (logMAR 0.097)
	Left Eye	20/22.5 (logMAR 0.05)	20/22.5 (logMAR 0.05)	20/30 (logMAR 0.18)	20/25 (logMAR 0.097)
6%	Right Eye	20/40 (logMAR 0.3)	20/33 (logMAR 0.2)	20/28.5 (logMAR 0.15)	20/30 (logMAR 0.18)
	Left Eye	20/22.5 (logMAR 0.05)	20/25 (logMAR 0.097)	20/30 (logMAR 0.18)	20/30 (logMAR 0.18)

Photic phenomena and patients satisfaction

In terms of photic phenomena, two out of four patients reported some sort of night driving difficulty, two patients reported haloes, but none of them reported experiencing glare or image distortion. Two out of four patients also reported having to increase their mobile screen brightness after the surgery to read better on their smartphones, but none of them reported having to increase the font size on their devices in order to do so.

Despite these occasional photic phenomena, all patients expressed their satisfaction with the quality of vision and the outcomes of surgery, especially since they all achieved spectacle independence at all distances. All patients indicated that they would recommend the procedure to a friend or a family member and on a scale from 1 to 10 (10 being completely satisfied with the results), three patients rated their satisfaction as 10/10 and one patient rated it as 9/10. Table 4 details the patients’ responses when answering a questionnaire about their experience with photic phenomena and their overall satisfaction.

**Table 4:** Patients’ responses to a questionnaire regarding their experience with photic phenomena and their overall satisfaction Never: The patient never experiences such symptoms; Occasionally: The patient experiences these symptoms one to three times a week; Often: The patient experiences these symptoms four to seven times a week; Always: The patient experiences these symptoms everyday, all the time

Questions	Case 1	Case 2	Case 3	Case 4
Do you experience difficulty with vision while driving at night?	Yes	No	Yes	No
How often do you experience haloes? (Never, Occasionally, Often, Always)	Often	Never	Occasionally	Never
How often do you experience glare? (Never, Occasionally, Often, Always)	Never	Never	Never	Never
How often do you experience image distortion? (Never, Occasionally, Often, Always)	Never	Never	Never	Never
Do you have to increase the screen brightness to read on your phone?	No	Yes	Yes	No
Do you have to increase the font size to read on your phone?	No	No	No	No
Since the surgery, have you ever needed glasses for “far” distance?	No	No	No	No
Since the surgery, have you ever needed glasses for “intermediate” distance?	No	No	No	No
Since the surgery, have you ever needed glasses for “near” distance?	No	No	No	No
Would you recommend the surgery to a friend or a family member?	Yes	Yes	Yes	Yes
From 1 to 10, what score would you give your overall satisfaction?	9	10	10	10

## Discussion

Cataract surgery in cases with stable keratoconus has always been challenging. While premium IOLs were generally avoided in the past with such cases, very encouraging results have been reported recently with the use of premium IOLs in selected cases of keratoconus, with acceptable results [6-15].

Our results show excellent outcomes in terms of visual acuity in all distances, contrast sensitivity, and overall satisfaction with quality of vision, all of which are consistent with previous results reported by Montano et al. in a case series that also included eyes with forme fruste keratoconus [14]. Compared to those included in the study published by Farideh et al. [15], cases included in our study had less severe topographic changes. Their study was conducted on eyes with frank, but mild, keratoconus.

It was interesting to find that although six out of the eight eyes included in our study had values of root mean square of corneal higher order aberrations (RMS-HOA) between 0.738µm and 1.27µm in the 6mm pupil, which are considered to be higher than those of the normal population, and higher than the 0.5µm cut-off value that is usually recommended by surgeons for multifocal IOL candidates [19,20], these patients did not experience significant haloes, glare or deterioration in the quality of vision as would have been expected. In fact, the patient in Case 1, who had total corneal RMS-HOA below 0.5µm (0.386µm in the right eye and 0.475µm in the left eye), was the one who reported experiencing haloes often in addition to difficulty driving at night.

These findings could suggest that despite the role that corneal HOAs play in exaggerating the photic phenomena experienced by some patients after receiving trifocal intraocular implants, patients with stable subclinical keratoconus who have been living with these high values of corneal HOAs all their lives may have developed a tolerance to HOAs that would render a relatively small increase in ocular HOAs that could occur after trifocal IOL implantation unremarkable to their quality of vision. Further studies with larger sample sizes comparing ocular HOAs before and after diffractive trifocal IOLs implantation in normal eyes and in those with high levels of corneal HOAs are necessary to confirm these findings.

Patients with stable keratoconus usually have limited options when it comes to presbyopia-correcting IOLs after cataract surgery. We believe that our results, and the results of previous reports, should encourage cataract surgeons in the future to consider diffractive trifocal IOLs for patients with stable subclinical or mild keratoconus, especially those who are keen on spectacle independence. We also maintain that such cases should be evaluated on a case-to-case basis, carefully studying each patient’s corneal tomography and HOAs as well as their personality and expectations in order to select those suitable for premium IOLs, all the while ensuring their full understanding of the drawbacks of such technology.

## Conclusions

Both AT LISA tri 839MP and AT LISA tri-toric 939MP have shown good post-operative outcomes in patients with stable subclinical and forme fruste keratoconus, with all patients achieving spectacle independence at all distances, reporting good quality of vision and high satisfaction rate, even in patients with higher than normal corneal RMS-HOA values. Our case series highlights the possibility of offering premium IOL options for patients with stable subclinical keratoconus when undergoing cataract surgery. However, careful patient selection remains an important factor in achieving favorable outcomes in such cases. 
